# Impact of an outdoor loose parts play intervention on Nova Scotian preschoolers’ physical literacy: a mixed-methods randomized controlled trial

**DOI:** 10.1186/s12889-023-16030-x

**Published:** 2023-06-13

**Authors:** Hilary A. T. Caldwell, Rebecca A. Spencer, Nila Joshi, Karina Branje, Jane Cawley, Heather Hobson, Sara F. L. Kirk, Daniel Stevens, Michelle R. Stone

**Affiliations:** 1 Healthy Populations Institute, Dalhousie University, 1318 Robie Street, Box 15000, Halifax, NS B3H 4R2 Canada; 2 School of Health and Human Performance, Dalhousie University, PO Box 15000, Halifax, NS B3H 4R2 Canada; 3 Interdisciplinary Studies, Dalhousie University, 6299 South Street, Halifax, NS B3H 4R2 Canada; 4 Department of Pediatrics, Dalhousie University, IWK Health Centre, 5850 University Avenue, PO Boc 9700, Halifax, NS B3K 6R8 Canada

**Keywords:** Childcare, Physical activity, Thematic analysis, Unstructured play, Early childhood education

## Abstract

**Background:**

Physical activity participation among preschoolers in childcare settings are low, and interventions to increase physical activity levels have produced mixed results. The Physical Literacy in the Early Years (PLEY) project implemented a six-month childcare-based outdoor loose parts play intervention in childcare centres in Nova Scotia, Canada. The purpose of this study was to examine the impact of the PLEY project on the development of domains of physical literacy (physical activity, physical competence, confidence and motivation, knowledge and understanding) in preschoolers attending childcare centres using mixed-methods.

**Methods:**

Preschoolers (3–5 years) were recruited from 19 childcare centres in Nova Scotia and centres were randomized (parallel design) to the outdoor loose parts play intervention group (*n* = 11) or control (*n* = 8) group for 6 months. Participants, early childhood educators, and assessors were not blinded to group assignment. Quantitative and qualitative measures were used to comprehensively assess the impact of the PLEY project on all domains of physical literacy. At 3- and 6-months, early childhood educators participated in focus groups to assess how the intervention supported the development of 4 physical literacy domains: physical activity, physical competence, confidence and motivation, and knowledge and understanding. Physical activity and physical competence were also assessed with accelerometry and the Test of Gross Motor Development-3, respectively.

**Results:**

Two hundred and nine preschoolers participated in the study (intervention group: *n* = 115; control group: *n* = 94)*.* Accelerometer data showed that while baseline physical activity was similar between groups, children in the intervention group had higher physical activity at 3- (F(1,187) = 8.30, *p* = 0.004) and 6-months (F(1,187) = 9.90, *p* = 0.002) post-intervention. There was no intervention effect on physical competence scores. Thematic analysis of focus group data revealed that outdoor loose parts play contributed to development in all 4 physical literacy domains, including increased movement repertoires, social development, and enjoyment of physical activity. No adverse events or side effects of the intervention were reported.

**Conclusions:**

Participation in the PLEY project was associated with increased development of various domains of physical literacy and perceived physical literacy among preschoolers, and outdoor loose parts play may be encouraged as an effective strategy to increase physical literacy in early learning settings.

**Trial registration:**

Biomed Central (ISRCTN14058106), 20/10/2017.

**Supplementary Information:**

The online version contains supplementary material available at 10.1186/s12889-023-16030-x.

## Introduction

Participation in regular physical activity in the early years (0–4 years) is associated with numerous physical, mental and social health benefits such as favourable motor skill and cognitive development, cardiometabolic health, fitness and psychosocial health [1]. Physical activity patterns differ greatly year to year in early childhood, suggesting patterns are not yet set in the early years [2]. Physical literacy, defined as “the motivation, confidence, physical competence, knowledge and understanding to value and take responsibility for engagement in physical activities for life” [3], describes the necessary elements for children to be active for life. Physical literacy literature commonly divides the concept into four, essential and interconnected domains (physical activity participation, physical competence, motivation and confidence, and knowledge and understanding) that develop across the lifespan and collectively contribute to an individual’s physical literacy [4, 5]. Physical activity promotion in early childhood should focus on developing physical literacy to ensure children are developing all the necessary ingredients for an active future.

The majority of Canadian toddlers and preschoolers are in some form of childcare arrangement and such environments provide a unique setting for physical activity promotion in the early years [6]. In childcare settings, preschoolers take part in low levels of total physical activity (TPA) and moderate-to-vigorous physical activity (MVPA) and high levels of sedentary time [7]. Preschoolers are generally more physically active when outdoors at childcare settings versus indoors [8]. Despite this, research suggests that allocating additional time to outdoor play only results in minimal increases in physical activity and additional efforts, such as portable equipment, are needed to significantly increase outdoor physical activity levels [9, 10]. Previous literature suggests that innovative strategies, such as activity rooted in physical literacy, may help increase physical activity levels in early childhood. For example, Cairney et al. proposed that physical literacy-based interventions be applied in childcare settings to target cognitive development because physical literacy extends beyond movement and additionally focuses on the fun and motivation of being active [11]. Given the multiple elements of physical literacy (affective, behavioural, cognitive, and physical), it is challenging to measure. In children and youth, several assessment batteries are available: Passport for Life, Physical Literacy Assessment for Youth (PLAY Tools) and the Canadian Assessment of Physical Literacy (CAPL) [12–14]. At the time of this study, there were no available tools to assess physical literacy in toddlers and preschoolers. However, without a specific physical literacy assessment tool, many studies have used a combination of tools as proxy measures for the multiple elements of physical literacy [15, 16].

To date, interventions to target physical literacy through outdoor play in childcare settings have been limited, but there is growing consensus about the importance of physical literacy-based physical activity opportunities for young children. Physical literacy experts have recommended that physical literacy-based interventions for preschoolers include opportunities for children to engage in free and outdoor play [17]. The Physical Literacy in the Early Years (PLEY) intervention was a mixed-methods randomized controlled trial that embedded loose parts into the outdoor play spaces of childcare centres across Nova Scotia from 2016–2018. ﻿As described in the PLEY project protocol paper, the goals of the PLEY project were to: (1) improve children’s physical literacy and increase time in physical activity and outdoor play during regularly scheduled outdoor time; (2) improve educators’ attitudes, beliefs, perceived competency, and intentions towards incorporating the intervention into practice; and (3) increase parents’ and educators’ understanding of play in child health and development [18]. We recently reported that there was a non-intervention effect on quantitative measures of preschoolers’ fundamental movement skills; however, educators spoke about how outdoor loose parts play provided opportunities for children to combine and repeat movements, and take risks, supporting physical, cognitive and socio-emotional development [19]. While some results of the PLEY project have been published [19–22], we have not yet examined if participating in the PLEY intervention was associated with increased physical literacy, as hypothesized.

The objective of this study is to explore the role of the PLEY project on Nova Scotian preschoolers’ domains of physical literacy (physical activity, physical competence, confidence and motivation, knowledge and understanding) using mixed-methods. A mixed-method approach allows us to build on previous reports of quantitative findings [19, 23] and provide further insight into how outdoor loose parts play (OLPP) in the PLEY project contributes to the development of physical literacy. In this study, physical literacy was conceptualized based on the International Physical Literacy Association’s definition that includes physical, affective, motivational and behavioural domains and has also been endorsed as Canada’s Consensus Statement on Physical Literacy [3, 4].

## Methods

### Study design

**Fig. 1:**
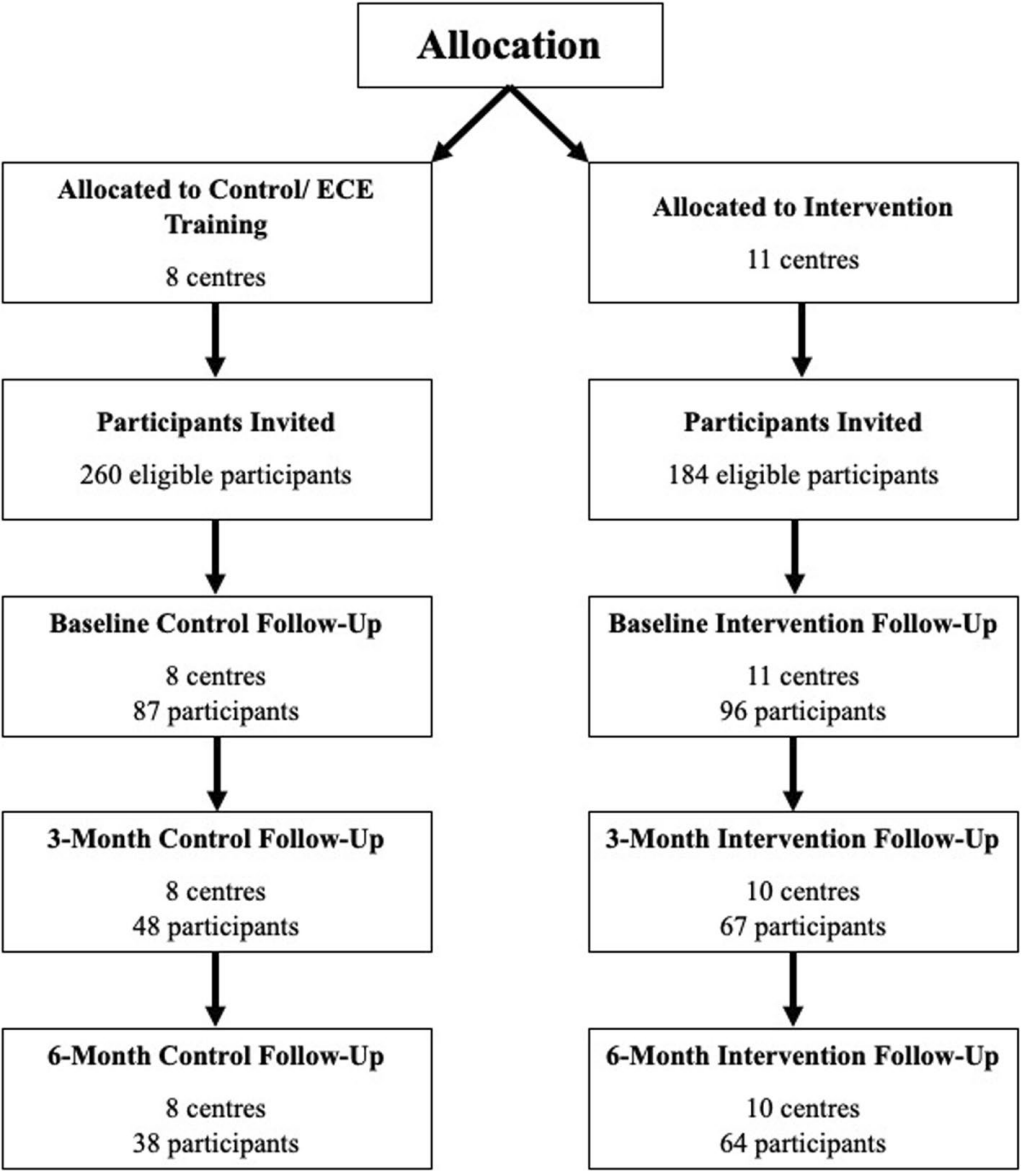
Flow diagram of participants (preschoolers) through the study

This mixed-methods study used a convergent parallel design [24] to collect both quantitative and qualitative data as part of the Physical Literacy in the Early Years (PLEY) project described previously in the protocol paper [18]. The PLEY project was a large parallel clustered randomized controlled trial conducted in Nova Scotian preschoolers aged 3 to 5 years that aimed to improve physical literacy, physical activity, and active outdoor play through the integration of OLPP at regulated provincial childcare centres. Data collection occurred from April 2016 to September 2018. OLPP was implemented for 6 months at intervention sites with data collection at baseline, 3-months, and 6-months (post-intervention). After recruitment and baseline assessments, centres were randomly assigned to the control or intervention group through computer based random number selections, based on rural and urban locations dispersed between the groups (see Fig. 1).

### Recruitment

The study and associated protocols were approved by the Dalhousie University Research Ethics Board (REB #2016–3924) and registered as a randomized controlled trial with Biomed Central (ISRCTN14058106; 20/10/2017). Informed consent forms were received from parents/ legal guardians of participating children, and from all participating educators. All methods were carried out in accordance with relevant guidelines and regulations, including with the REB approved protocols. All Nova Scotia licenced childcare centres with an enrolment of greater than 20 children aged 3–5 years were sent a general inquiry of interest by email. All interested sites then received an in-person site visit to further discuss participation in the project from a member of our project team who had work experience as an early childhood educator and significant knowledge of and familiarity with the childcare sector. Twenty-one sites expressed interest; however, 2 were excluded as they were already advanced in their implementation of loose parts. The study included 19 childcare sites (parallel design; intervention: *n* = 11; control: *n* = 8). Sixteen sites (intervention: *n* = 8; control: *n* = 8) were initially recruited in November/ December 2016 and then randomized to the intervention or control group using random number generation. Three additional sites were recruited for the intervention condition in November 2017 to account for the drop-out of 1 centre (October of 2017), and to account for participant (child) withdrawal. Due to the timing of data collection, many of the recruited 4-year old participants left the childcare centres in September to attend the newly-established Nova Scotia pre-primary program and additional centres and participants allowed our study to maintain its intended sample size. All children aged 3 to 5 years attending participating childcare centres were eligible to participate in the study; however, assessments were only completed with children whose parents provided written consent. All educators from intervention centres were invited to participate in the focus groups. Additional recruitment details are included in the PLEY project’s protocol paper [18].

### Intervention

The PLEY project used a socio-ecological approach to address preschoolers’ physical activity, physical literacy, and outdoor play at multiple levels of influence, including intrapersonal, interpersonal, organizational, community, and physical environment [18, 25]. The PLEY project intervention components included: a 6.5 h education session for educators (delivered by the research team) and loose parts kits for each intervention site. The intervention did not focus on added outdoor time for children. The education sessions taught educators about the importance of unstructured, child-directed play, and the value of loose parts, fundamental movement skills, physical literacy, and risky play for children’s health and development. The loose parts kits included buckets and lids, rope and a pully, tree cookies (slices of logs), milk crates, a package of hose tube, 20 + balls of a variety of sizes and weights, wood pieces, bread tray, large cardboard tubes, funnels of different sizes, a tarp, 5’ planks, 5’ PVC tubing (4″ and 2″ diameter), rocks, and tires [18]. The educators were instructed to provide the loose parts to children during all outdoor play sessions for the duration of the study (6 months). The control sites were asked to continue their regular outdoor play programming for the duration of the study, and received a loose parts kit at the end of the study (following final data collection).

### Assessment of physical literacy

At the time of data collection for the PLEY project, assessment tools to specifically measure preschoolers’ physical literacy were not available. As a result, the PLEY project conceptualized the assessment of physical literacy based on the term’s definition [3], similar to methods used in previous work with youth [26] and young adults [27]. The outcome assessors were not blinded to the intervention and control sites due to resource limitations and it would not have been possible to blind outcome assessors at the 3- or 6-month timepoints as all assessments were completed at the centres and loose parts would have been visible to the assessors. For the present study, daily physical activity behaviour is represented as device-measured physical activity and educator focus group data about physical activity. The physical competence domain is reflected as fundamental movements skills (FMS) and educator focus group data about physical competence. Confidence and motivation and knowledge and understanding domains were captured with educator focus groups. Some physical activity quantitative data [23] and all physical competence quantitative data [19] have been reported previously. The publication serves to collectively report on the impact of the PLEY project on the development of physical literacy by combining quantitative and qualitative results using mixed-methods.

#### Accelerometry (physical activity domain)

Physical activity data collection and analysis methods have been described previously [22]. Briefly, children in the intervention and control groups were asked to wear the accelerometers (ActiGraph wGT3X + ; ActiGraph, LLC, Pensacola, FL, USA) during waking hours for 9 consecutive days at the three time points (baseline, 3-months, and 6-months). Data were collected in 15 s epochs, and non-wear time was defined as 20 min or more of consecutive zero counts [28]. To be included in the analysis children required at least 4 days (childcare days only) with at least 6 h of valid wear time each day [29]. Previously published cut-points to establish intensity thresholds were used for this investigation (total physical activity (TPA): > 100–1679 counts/min; moderate-to-vigorous physical activity (MVPA): ≥ 1680 counts/min) [30], and accelerometer data were specifically analyzed for the childcare period, which was defined as 7:30 AM to 5:30 PM. TPA and MVPA are expressed as minutes/day.

#### Test of Gross-Motor Development-3 (physical competence domain)

Data collection for FMS data has been described previously [18, 19]. Briefly, participants in both the intervention and control groups completed an assessment of FMS at baseline (1–3 months prior to the intervention), and following the introduction of loose parts to intervention sites (3- and 6-month time points). The Test of Gross-Motor Development-3 (TGMD-3), a validated tool for children from birth to 5 years of age, was used to evaluate FMS [31, 32]. A sum of all locomotor skills and object control skills was used to calculate a total FMS score [31]. Balance was assessed with the Preschooler Gross Motor Quality Scale (PGMQ) and a total balance score was calculated [33].

#### Educator focus groups (all physical literacy domains)

Fifteen focus groups took place (9 at 3-months and 6 at 6-months), with 3–5 participants in each group. The focus groups included educators from multiple sites and took place in public locations. Educators from all intervention sites were represented in the focus groups. The focus groups included a series of questions divided into several categories: active outdoor play, loose parts, risk-taking, policies, and challenges/benefits of the intervention. For example, educators were asked “what happened when loose parts were introduced in the outdoor environment for the children?”, “describe any changes you may have seen in the children’s development—social, cognitive, physical, emotional, or others”, and “what do you do when children are playing outside?”. These focus groups, which lasted approximately 45 to 60 min, allowed for more in-depth exploration of what was challenging and/or what was helpful to educators in using the loose parts in their daily activities. The focus group methodology has been described previously [18–21]. The data from educator focus groups provided additional context and narrative to the objective measures of physical activity and physical competence.

### Data analysis

#### Quantitative

TGMD-3, balance assessment and accelerometry data were analysed and described in detail previously [19, 22, 23]. Briefly, changes in FMS and physical activity between children at control and intervention sites were examined using multilevel modelling for repeated measures with intention-to-treat analysis (SAS University Edition, SAS Institute, Inc, Cary, NC). Multilevel modeling accounted for possible clustering within childcare centres. Possible confounding variables, such as age, sex, body mass index and socioeconomic status were included in the models. 95% confidence intervals (95% CI) and p-values were calculated, and statistical significance was defined as an alpha less than 0.05. It was previously determined that a sample size of 180 would be sufficient to have an 80% chance for detecting a 10% difference in physical literacy between the composite scores of the intervention and control group at the 5% significance level and for selecting moderate between-group effects in FMS [18, 34].

#### Qualitative

**Fig. 2:**
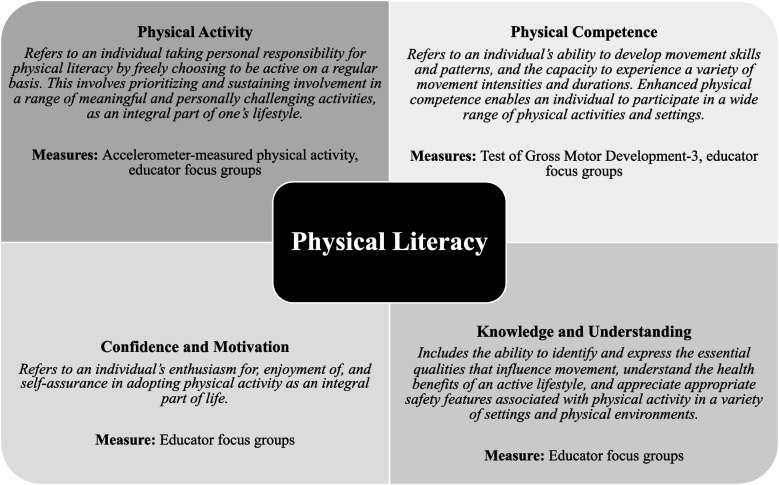
Theoretical model for the assessment of physical literacy in the Physical Literacy in the Early Years project (physical literacy domains adapted from Canadian Physical Literacy Consensus Statement, 2015)

Analysis of focus group data has been described previously [19, 20]. Focus group content was analyzed using thematic analysis, and themes were identified within each of the physical literacy domains [35, 36]. Briefly, data were audio-recorded and transcribed verbatim. Data were organized using Microsoft Word and imported into QSR NVivo 11 for analysis. Data analysis was conducted primarily by research staff and guided by a senior member of the research team. Transcripts were coded using deductive coding based on physical literacy domains (Fig. 2). Regular meetings were held to discuss codes and develop a codebook. Analyses of quotes within each physical literacy domain was guided by thematic analysis using a collaborative process by which relationships between codes and trends in the data were identified and discussed [35]. Final themes were agreed upon by the research team. Focus group data were also coded for different movement skills mentioned as an indicator of increased movement repertoire within the physical competence domain. Movement skills were categorized as locomotor, object control, or balance skills to match the domains measured objectively with the TGMD-3 [31].

## Results

Results are presented within the four domains of physical literacy (physical activity, physical competence, confidence and motivation, knowledge and understanding). Participant flow through the study is outlined in Fig. 1. Table 1 includes the FMS and accelerometry results at baseline, 3- and 6-months for participants in the control and intervention groups. Descriptive statistics are presented as mean (standard deviation) for continuous variables or as *n*(%) for categorical variables. At baseline, 209 children participated (4.2 ± 0.6 years, 44.7% girls, 16.1 ± 1.4 m/kg^2^). Themes within each physical literacy domain that were identified through thematic analysis of educator focus group data are summarized in Table 2. No harms or adverse events were reported during the intervention.

**Table 1:** Results of quantitative assessments of physical activity and fundamental movement skills

	Baseline	3-Month	6-Month
Total Physical Activity (minutes/day)			
Control^b,d^	374 (34)	396 (35)^a^	395 (38)^a^
Intervention^b,d^	354 (30)	458 (34)	430 (31)
Moderate-to-vigorous physical activity (minutes/day)			
Control^b,d^	180 (34)	198 (30)	203 (32)
Intervention^b.c^	170 (29)	188 (28)	175 (22)
*Total Fundamental Movement Skills*			
Control^b,c,d^	47.5 (12.9)	54.0 (11.8)	58.6 (14.5)
Intervention^b,c,d^	49.3 (15.5)	56.3 (8.4)	60.3 (12.5)

Values presented as mean (standard deviation)

^a^significant difference between control and intervention groups

^b^significant change between baseline and 3 months within groups

^c^significant change between 3 and 6 months within groups

^d^significant change between baseline and 6 months within groups

**Table 2:** Summary of domains and themes identified in educator focus groups

**Physical Literacy Domain**		**Themes**
Physical activity	1	OLPP contributed to physical activity
Physical competence	2	OLPP contributed to increased physical competence
	3	OLPP contributed to an increased movement repertoire
Confidence and motivation	4	OLPP increased confidence in physical abilities and desire to try new or challenging activities
	5	OLPP increased enjoyment of physical activity
Knowledge and understanding	6	OLPP increased knowledge/learning about physical activity
	7	OLPP contributed to increased cognitive and social development

*OLPP* outdoor loose parts play

### Physical activity domain

#### Quantitative

Valid accelerometry data were available for 130 preschoolers at baseline (67% adherence), 71 at 3 months, and 62 at 6 months. MVPA and TPA had curvilinear relationships over time, with MVPA and TPA increasing from baseline to 3 months and decreasing from 3 to 6 months (Table 1). Within groups, both TPA and MVPA were higher at 3 months compared to baseline. TPA was higher at 6 months compared to baseline in both the intervention and control groups. MVPA was higher at 6 months compared to baseline in the control group, whereas MVPA increased from baseline to 3-months and then declined form 3-months to 6-months in the intervention group. There was a statistically significant group-by-time effect of TPA such that TPA was similar between groups at baseline, and higher in children in the intervention group at 3 (F(1,187) = 8.30, *p* = 0.004) and 6 months (F(1,187) = 9.90, *p* = 0.002). There were no overall group-by-time- differences for MVPA (*p* > 0.05).

#### Qualitative

### Theme 1: OLPP contributed to increased physical activity

In focus groups, educators reported that they observed that OLPP contributed to increased physical activity levels during outdoor play sessions; for example, one educator commented “*they were active, they were more active…*” and another said “…*they’re getting a lot of that activity with us…*”. It was also observed that OLPP may have been particularly useful to increase physical activity in less active children, with one educator saying “…*like some children who were very physically active, were very physical players in the beginning, it didn’t change for them, they still were very physical, but the loose parts maybe gave an opportunity for the children who weren’t always so physical a way to do that*”. The educators also commented that OLPP was an opportunity for children to take part in unstructured physical activity, versus the primarily structured physical activity opportunities provided at home and by parents; for example, one educator suggested that educators could play a role in encouraging more family physical activity at home by saying “*there’s time, it is how you’re using your time right, maybe limited time at the end of the day if they’re picking up their kids late, but on the weekends, particularly, could we be encouraging loose parts play in the home on the weekend, getting away from more of the structured activities, into the unstructured family-centered play*”. In all, educators shared that the OLPP intervention contributed to increased unstructured physical activity while at childcare and that this could be expanded to children’s home environments as well.

### Physical competence domain

#### Quantitative

As we previously reported, there was no intervention effect on any of the FMS variables. All FMS variables increased across the three time points in children attending intervention or control sites [19].

#### Qualitative

### Theme 2: OLPP contributed to increased physical competence

Several educators commented in the interviews how they observed that children’s physical competence was developing and improving over the course of the intervention; for example, they said: “*they’re much, much, much, much, more competent…*”. One educator commented that they were not initially drawn to the concept of physical literacy until they observed the children developing and playing with loose parts, “*I’ll admit it wasn’t initially like the physical literacy thing that drew me in, it was like the problem solving, it was the cooperation, seeing how they were working together, to help each other off the slide and then when they figured out how to get it around on the other side, I was like that’s really cool but then when I started watching I noticed how they were using all these physical skills as well*.” In addition, the loose parts may have provided an extra benefit to children with lower physical competence, as one educator said “*…there’s a boy in particular who his muscle development was not quite there, who was definitely behind his peers and his parents had mentioned that he had come a long way as well with all of those things and he was enjoying the experience of having all those different things to do out there and that really helped him a lot and it helped some of the children who are not as coordinated”.*

The increased physical competence that educators observed was primarily developed through multiple attempts or trial-and-error. As one educator shared, *“some of them are stronger, like some of them will struggle with it and then a week later you’ll see them and they’re just sailing down the playground with whatever they couldn’t do before…”* and another commented how the children develop competence very quickly, “*and then the very next day, he was able to master the skill he wasn’t able to do the day before*”*.* Educators were supportive of this development and encouraged children to keep trying something, even if it was difficult, “*…we tell them you know they can do this if you are able to, your body is able to do it, eventually yeah like sometimes they might try you know a couple of days or a week, a few months, then all of a sudden they’re able to do something, and it’s like I did it, and it’s that, it is, it’s a sense of pride, it’s a sense of accomplishment…”.* In summary, educators perceived that the PLEY project provided opportunities for children to develop their physical competence through unstructured, child-led, OLPP.

### Theme 3: OLPP contributed to an increased movement repertoire

Educators shared how OLPP contributed to an increased movement repertoire, as evidenced by one educator’s comment, “*a lot of different movements, a lot of muscles being used*”. The educators shared the wide range of movements and skills that children were using, including both skills captured in the TGMD-3, such as throwing and hopping, and skills not commonly captured in FMS assessments, such as climbing or dragging. While educators were not specifically asked which movements they observed, any movement skills mentioned in the focus groups was coded. Table 3 includes the numerous movement skills that educators observed and shared during the educator focus groups, and subsequently organized as locomotor, object control or balance skills, the same categories of movements captured in the quantitative assessments. Educators identified the many ways children were climbing; for example, “*it was quite a high playhouse and they would climb on it*” and *“…yesterday they were climbing the tree with the rope that was provided*”. In some cases, the climbing was combined with other movements like crawling, “*and they would have to, you know, crawl along, on their hands and knees and then they were underneath um, their stomach and kind of shimmying underneath and climbing through, like this, on their stomach*”. Children also used object control skills when playing with loose parts that are not captured in traditional motor skill assessments, such as dragging, pulling, and pushing. For example, children used a variety of movements to move larger or heavier loose parts, *“…picked a bag of kindling up, and he threw it over his shoulder and he walked across the playground…*”, “*…and so this little boy found a plank and he kind of picked up one end of it and dragged it over to the house”* and “*they were pulling things with a rope*”. It was also shared that children were using balance in their OLPP, including balancing on objects such as planks, “*they were balancing, they were trying to keep control of the board like from moving from side to side and trying not to fall off…”.* Children were also building structures and then balancing on what they built, as one educator commented, “*We have very good stumps on our playground so they went and got one of the great big ones and they had one of the planks so they put it in the middle, they balanced on it, but then they were trying to figure out how to get it to actually balance from standing on it so they were problem solving*”.

**Table 3:** Movement skills identified by educators in the educator focus groups

Locomotor skills	Object control skills	Balance skills
Climb	Bounce ball	Balance (general)
Crawl	Bury object	Balance on surface (e.g., plank)
Dance	Catch/ receive object^a^	
Jump^a^	Carry object	Balance while moving^a^
Run^a^	Collect/ gather objects	
Reach/ stretch	Dig	
Skip^a^	Drag object	
Slide^a^	Kick^a^	
Swing	Lift/ pick-up object	
Walk	Pile/ stack object	
	Push object	
	Roll object	
	Throw object^a^	

^a^movement skills also captured in the quantitative assessments of fundamental movement skills

### Confidence and motivation domain

#### Qualitative

### Theme 4: OLPP increased confidence in physical abilities and desire to try new or challenging activities

In the focus groups, educators repeatedly shared how the loose parts helped increase children’s confidence in their physical abilities. For example, one educator shared: “*I don’t know if it’s necessarily actually like their strength or their ability, I think the confidence in their abilities is so much stronger that even if they were able to do it before, they wouldn’t necessarily try to do it*”. Educators shared that with regular exposure to the loose parts, preschoolers were becoming more confident in skills: “*so like if they were doing something like this [before the loose parts], they would have maybe walked really slow before and now they’re like almost like speed walking across and they’re like no I can do this, like more confident in themselves”.* Educators also observed that children became aware of their comfort levels with different activities as they became confident in their physical abilities, *“…they tend to have a better sense of what they feel comfortable doing and often they won’t do something if they’re not actually ready for it. Like I’ll have some children who have literally done things that like will sometimes stop my heart, like they’re the daredevils, but then there’s the other ones that know ‘okay I’m not ready for that yet so I’m not going to jump from up here, I might try jumping from this ledge cause it’s more comfortable for me’. So I think it’s also just trusting that they know where they’re at*”. The increased confidence in physical abilities was also linked to an increased motivation to play, take risks, and challenge themselves.

Coupled with the increased confidence in physical abilities, educators shared that children had increased confidence and motivation to try something challenging or risky once exposed to OLPP. As one educator shared, “*they were more eager to take risks, to – like you know what I mean, like after using these materials in different ways, they were more eager to – whereas the first day – it was just kids lifting them up and looking at them…*”. In another example, an educator observed that children were confident to try to walk across narrower planks when thicker ones were available, “*I think there were a couple of times where she went, I was not encouraging her but saying that there may be other planks or whatever and she was like ‘no I don’t want the wider planks, I want the smaller ones’ and that was all her because I know that obviously the thicker plank would have been easier for her to walk across but she didn’t want it, she was determined to do the thin, little, tiny 1 inch ones*”. The increased confidence in their ability to walk on narrower planks translated into the motivation to do the more challenging tasks. Educators shared that children who were previously more fearful were more confident to try more challenging things, “*I feel that the ones who were fearful, who were not likely to get up on something and walk across something or are far more likely to do something like that now…”.* The educators also adapted and supported the children in their more challenging or risky play, *“…you can see the older they get all of a sudden they just become very brave jumpers and we try to keep it safe, you know limit where they can jump, where we know it might not be safe and let them jump where we know it is safe, and let them go*”.

### Theme 5: OLPP increased enjoyment of physical activity

The second theme related to confidence and motivation highlighted the children’s enjoyment to engage in OLPP over the course of the intervention. One educator shared how they also enjoyed the loose parts, *“…after a while, I could tell they really warmed up to the idea, and they really loved the loose parts, they really enjoyed them. And um, really, it’s kind of, it has converted me, you know, [yeah] and I would love to, get more- more, you know, involved with loose parts idea”*. Educators felt that children who were more timid really enjoyed playing and engaging with the loose parts, “*exactly – I was going to say – even the children who – again, are more timid, yeah, they’re just – you can see that they’re really enjoying it… they really taken this idea and they’ve just run with it. I’ll – it’s been wonderful*”.

### Knowledge and understanding domain

#### Qualitative

### Theme 6: OLPP increased knowledge/learning about physical activity

Educators perceived that much of the children’s learning with OLPP happened through mimicry, modelling, and peer leadership, as children observed other children do something and would then proceed to try it themselves, or with guidance from their peers. This quote displays that educators perceived children were learning by playing with one another, without the involvement of their educators: “*well the other kids were playing…so the other kids were like watching them, like what are they doing and then some of them came over to try it….”.* By engaging in OLPP, educators observed that children demonstrated their leadership abilities: “*he was kind of manager of the project. And they were all helping, and it was a very collaborative effort…”,* and younger children were learning from the older children. Similarly, one educator commented*, “so it was really nice to see how they worked together and were mentoring each other and cooperating and helping the younger ones…”*. Overall, educators perceived that OLPP fostered children’s learning and knowledge to further engage and play with loose parts, and provided opportunities for children to learn from one another.

### Theme 7: OLPP contributed to cognitive and social development

In addition to increased knowledge and learning about PA through OLPP, educators observed that the OLPP helped with children’s cognitive and social development, including teamwork, collaboration, problem solving skills, and independence. One of the educators shared what they believed children developed through OLPP: “*teamwork, and turn taking, and encouraging each other, sharing ideas, um, building confidence through the whole thing. You know, really learning, um, independence –play and, um, learning to, uh – or coach each other for what to do next and then feed from each other*”. Several comments suggested the children played collaboratively with loose parts and were able to problem solve without the support of adults, “*Oh just when it was higher like I think the crates falling over. They’re pretty lightweight, they’re fairly easy to carry so and they’re pretty good with telling their friends to watch it, we need more space*” and another educator shared “*Like my group of four year olds collectively decided where’s the path, where is it too high, like my, like the class independently decided that’s too high, we should not jump from here, and they did that independently cause they jumped off there like that hurts my feet when I land, that was their risk assessment*”. Collaboration and problem solving were also highlighted when an educator shared: *“…cause they would give directions, so they really understood the ways they had to balance you know heavy and light and what would make one go up and the other go down, like they understood that process, they didn’t use the words exactly but you can tell that they knew what they had to do*”. Independence during OLPP was also highlighted in the focus groups; for example, one educator said, “*well that’s just, like they don’t look for us nearly as much outside as they do inside*”.

## Discussion

This study described the role of the PLEY project’s OLPP intervention on domains of physical literacy in preschoolers attending childcare centres in Nova Scotia. Previous results from this study suggested that the OLPP intervention had a positive impact on some measures of physical activity and no impact on physical competence assessed with a traditional FMS assessment tool [19, 23]. From the perceptions of educators, we reported that the OLPP contributed to the positive development of physical literacy in four domains: physical activity, physical competence, confidence and motivation, and knowledge and understanding. The mixed methods used in this study were advantageous to provide a comprehensive assessment of the impact of the OLPP intervention on all aspects of preschoolers’ physical literacy.

While specific physical literacy assessments have been validated for use in school-age children [37, 38], a complementary assessment tool for preschoolers was not available when the PLEY project was implemented. For example, the Preschool Physical Literacy assessment tool was developed after the PLEY project began [39]. In the absence of a validated tool for preschoolers, we combined quantitative and qualitative measures to comprehensively capture the four domains of physical literacy. To our knowledge, this is the first study to describe the impact of an OLPP intervention on all domains of physical literacy among preschool children.

Children in both the intervention and control groups had increases in MVPA and TPA from baseline to 3 months; and levels then slightly decreased from 3 to 6 months. At both 3 and 6 months, TPA, but not MVPA, was higher among children in the intervention versus control group. Despite these results, educators in the intervention group perceived children to be more active during outdoor play once loose parts were introduced. Similarly, preschoolers who took part in a childcare intervention that involved both structured and unstructured physical activities displayed greater increases in TPA, but not MVPA [40]. Interestingly, Tucker et al. (2017) observed an increase in MVPA and no change in TPA following an 8-week intervention in childcare settings that implemented staff training and portable play equipment, but that the positive impact on physical activity was not sustained at 6- or 12-month follow-up [41]. The reduction in physical activity from 3 to 6 months that we observed may be because the novelty of loose parts wore off after the initial excitement in the first few months. To mediate this effect in practice, program leaders could stagger the introduction of different loose parts regularly to maintain enthusiasm and excitement. It is important to note that physical activity levels were generally high in our sample and further increases were not possible as this intervention did not provide additional time for physical activity, but modified the outdoor environment. The generally high physical activity levels of our sample may be due to the children’s daily schedules in regulated childcare centres, or due to data reduction decisions, as outlined in our previous work [22].

Preschoolers’ physical competence was not improved based on a traditional FMS assessment following participation in the PLEY project [19], but educators reported that OLPP supported the development of preschoolers’ physical competence and increased their movement repertoires. FMS were assessed with the TGMD-3, a tool that assesses sport-related FMS such as running, kicking, and catching [31]. The PLEY OLPP intervention did not specifically target the development of these sport-specific FMS as the intervention did not include intentional skill-development sessions or coaching. The educators observed preschoolers developing physical competence as they played outdoors with loose parts and commented that the unstructured play environment allowed them to learn through trial-and-error. Educators also shared the large repertoire of movements they observed, including some of the FMS (run, jump, throw, kick) captured in the TGMD-3, as well as movements not captured in the assessments, such as climbing, rolling, or pushing objects. Our previous results also suggest preschoolers demonstrated various combinations of FMS when playing with loose parts, such as pulling and carrying objects, rather than performing movements in isolation [19].

Educators shared that OLPP positively impacted preschoolers’ confidence, specifically their confidence in their physical abilities and their desire to try new or challenging activities. Motivation and confidence is an important domain of physical literacy because it is essential that children are enthusiastic about and enjoy movement to prepare them for a lifetime of physical activity [4]. It has been proposed that physical literacy based interventions for young children target the development of confidence and positive affect by scaling activities to a child’s ability, ensuring there are opportunities for mastery, and allowing children to personalize activities—all characteristics included in the PLEY OLPP intervention [11]. Educators in the PLEY project also reported that loose parts enable children to take risks and help them become less fearful of active outdoor play, and that loose parts helped preschoolers to cultivate independence, confidence, self-esteem and pride [20]. It was also reported that the preschoolers were excited to go outside and play with the loose parts. Our findings align with previous literature that suggests loose parts is associated with increases in intra-personal enjoyment and co-operative play, and a higher odds of being happy at school [42]. Similarly, early childhood educators have reported that children improved socialization, creativity and self-confidence after being exposed to an intervention to increase opportunities for nature and risky play at childcare centres in Vancouver, Canada [43]. In our study, OLPP may have helped preschoolers develop the confidence and motivation to be active—feelings that can hopefully be maintained across a lifespan of physical activity.

Without direct measures of knowledge and understanding from the preschoolers, this domain was challenging to capture. However, educators provided important insights and observations during focus groups to help us understand how children developed their knowledge and understanding about physical activity. Due to the unstructured, child-led nature of the PLEY intervention, preschoolers learned by observing and mimicking each other’s movements and actions. Educators also reported how some children took on leadership roles when playing with the loose parts. Secondly, the educators observed that OLPP contributed to preschoolers’ increased cognitive and social development, particularly the development of teamwork, collaboration, and problem-solving skills. In a study that implemented natural risky play environments in childcare centres, early childhood educators also observed increases in children’s problem solving skills as they played in the updated play spaces [43]. Work is needed to understand how these findings contribute to a lifetime of physical activity.

Several strengths and limitations of the PLEY project have been described previously [19]. A major strength of this manuscript was the mixed-methods approach to assess how OLPP contributed to the development of all domains of physical literacy among preschoolers. Our study is strengthened by the novel implementation of loose parts in outdoor play settings. The inclusion of educators’ perspectives was particularly valuable as these professionals regularly interacted with and observed children and were able to provide comprehensive assessments of children’s development of physical literacy. This study was limited by the limited accelerometer data (67% adherence at baseline, 30% adherence at follow-up) and the lack of one overall physical literacy measure. Given the methods used, we can report the impact of OLPP on each domain of physical literacy but not overall physical literacy. We are also limited by the lack of individual, child-level measures of the confidence and motivation and knowledge and understanding domains of physical literacy and we plan to explore this in our future work. Due to changes in season and weather, children may have had different opportunities for outdoor play with the loose parts throughout the intervention. Due to resource and personnel limitations, the outcome assessors were members of the core study team and not blinded to the intervention or control sites and may have introduced some bias in their assessments. Lastly, it was not feasible to collect fidelity assessments as the burden would have been too high on educators for the duration of the study. Through focus groups, educators shared that preschoolers were playing with the loose parts but the exact frequency and duration of loose parts play is unknown.

## Conclusion

We observed that participation in the PLEY project was positively associated with the development of domains of physical literacy (physical activity, physical competence, confidence and motivation, knowledge and understanding) among a sample of preschoolers. Using a mixed-methods design, we reported that OLPP contributed to higher total levels of physical activity using both quantitative and qualitative data. There was no intervention effect on the quantitative assessments of preschoolers’ FMS. However, qualitative analyses of focus group data revealed that educators perceived OLPP contributed to the development of physical competence and increased movement repertoires. Educators also perceived that preschoolers became more confident and motivated to play outdoors, and that they started to develop the knowledge and understanding to be active for life. Future studies should consider the use of additional tools to quantitatively assess physical activity and physical competence in ways that measure the types of movements children use when engaging in OLPP, such as behavioural mapping or other direct observation techniques. Future work should continue to explore the use of mixed-methods (i.e., accelerometers and interviews/focus groups) to simultaneously capture the volume and context of physical activity participate in childcare settings. As more physical literacy-specific assessment batteries are developed and validated, it will be essential to use these tools in future work to align findings with other research in this field. Our findings suggest early learning settings may consider implementing OLPP as a novel strategy to support the development of all domains of physical literacy in young children. The addition of loose parts to outdoor play settings may be advantageous for the development of young children’s physical literacy, positioning them to be active for life.

## Supplementary Information

**Additional file 1.**

## Data Availability

The datasets analysed during the current study are available from the corresponding author on reasonable request.

## Abbreviations

CAPL: Canadian Assessment of Physical Literacy
CI: Confidence interval
FMS: Fundamental movement skills
MVPA: Moderate-to-vigorous physical activity
OLPP: Outdoor loose parts play
PGMQ: Preschooler Gross Motor Quality
PLAY: Physical Literacy Assessment for Youth
PLEY: Physical Literacy in the Early Years
REB: Research Ethics Board
TGMD: Test of Gross Motor Development
TPA: Total physical activity
