# Supplementary material for: Impact of an outdoor loose parts play intervention on Nova Scotian preschoolers’ physical literacy: a mixed-methods randomized controlled trial
Source: BMC Public Health. 2023 Jun 13;23:1126. doi: 10.1186/s12889-023-16030-x (PMC10262461; doi:10.1186/s12889-023-16030-x)
Supplement: Supplementary file 1 — Additional file 1. [file 12889_2023_16030_MOESM1_ESM.docx]

Appendix 1.

**Table 1**, Summary of focus groups themes and example quotes identified in educator focus groups

| **Theme** | **Example Quotes** |
| --- | --- |
| OLPP contributed to physical activity | - “They were active, they were more active…” - “…they’re getting a lot of that activity with us...” - “…like some children who were very physically active, were very physical players in the beginning, it didn’t change for them, they still were very physical, but the loose parts maybe gave an opportunity for the children who weren’t always so physical a way to do that” - “There’s time, it is how you’re using your time right, maybe limited time at the end of the day if they’re picking up their kids late, but on the weekends, particularly, could we be encouraging loose parts play in the home on the weekend, getting away from more of the structured activities, into the unstructured family-centered play” |
| OLPP contributed to increased physical competence | - “They’re much, much, much, much, more competent…” - “I’ll admit it wasn’t initially like the physical literacy thing that drew me in, it was like the problem solving, it was the cooperation, seeing how they were working together, to help each other off the slide and then when they figured out how to get it around on the other side, I was like that’s really cool but then when I started watching I noticed how they were using all these physical skills as well” - “…there’s a boy in particular who his muscle development was not quite there, who was definitely behind his peers and his parents had mentioned that he had come a long way as well with all of those things and he was enjoying the experience of having all those different things to do out there and that really helped him a lot and it helped some of the children who are not as coordinated” - “Some of them are stronger, like some of them will struggle with it and then a week later you’ll see them and they’re just sailing down the playground with whatever they couldn’t do before…” - “And then the very next day, he was able to master the skill he wasn’t able to do the day before” - “…we tell them you know they can do this if you are able to, your body is able to do it, eventually yeah like sometimes they might try you know a couple of days or a week, a few months, then all of a sudden they’re able to do something, and it’s like I did it, and it’s that, it is, it’s a sense of pride, it’s a sense of accomplishment…” |
| OLPP contributed to an increased movement repertoire | - See Table 3 for full inventory of movement skills identified in the educator interviews “A lot of different movements, a lot of muscles being used”. - “It was quite a high playhouse, and they would climb on it” - “…yesterday they were climbing the tree with the rope that was provided” - “And they would have to, you know, crawl along, on their hands and knees and then they were underneath um, their stomach and kind of shimmying underneath and climbing through, like this, on their stomach” - “…picked up bag of kindling up, and he threw it over his shoulder and he walked across the playground…” - “…and so this little boy found a plank and he kind of picked up one end of it and dragged it over to the house” - “They were pulling things with a rope” - “They were balancing, they were trying to keep control of the board like from moving from side to side and trying not to fall off…” - “We have very good stumps on our playground so they went and got one of the great big ones and they had one of the planks, so they put it in the middle, they balanced on it, but then they were trying to figure out how to get it to actually balance from standing on it so they were problem solving” |
| OLPP increased confidence in physical abilities and desire to try new or challenging activities | - “I don’t know if it’s necessarily actually like their strength or their ability, I think the confidence in their abilities is so much stronger that even if they were able to do it before, they wouldn’t necessarily try to do it” - “So, like if they were doing something like this, they would have maybe walked really slow before and now they’re like almost like speed walking across and they’re like no I can do this, like more confident in themselves” - “…they tend to have a better sense of what they feel comfortable doing and often they won’t do something if they’re not actually ready for it. Like I’ll have some children who have literally done things that like will sometimes stop my heart, like they’re the daredevils, but then there’s the other ones that know ‘okay I’m not ready for that yet so I’m not going to jump from up here, I might try jumping from this ledge cause it’s more comfortable for me’. So, I think it’s also just trusting that they know where they’re at” - “They were more eager to take risks, to – like you know what I mean, like after using these materials in different ways, they were more eager to – whereas the first day – it was just kids lifting them up and looking at them…” - “I think there were a couple of times where she went, I was not encouraging her but saying that there may be other planks or whatever and she was like ‘no I don’t want the wider planks, I want the smaller ones’ and that was all her because I know that obviously the thicker plank would have been easier for her to walk across but she didn’t want it, she was determined to do the thin, little, tiny 1 inch ones” - “I feel that the ones who were fearful, who were not likely to get up on something and walk across something or are far more likely to do something like that now…” - “…you can see the older they get all of a sudden they just become very brave jumpers and we try to keep it safe, you know limit where they can jump, where we know it might not be safe and let them jump where we know it is safe, and let them go” |
| OLPP increased enjoyment of physical activity | - “…after a while, I could tell they really warmed up to the idea, and they really loved the loose parts, they really enjoyed them. And um, really, it’s kind of, it has converted me, you know, [yeah] and I would love to get more- more, you know, involved with loose parts idea” - “Exactly – I was going to say – even the children who – again, are more timid, yeah, they’re just – you can see that they’re really enjoying it… they really taken this idea and they’ve just run with it. I’ll – it’s been wonderful” - “I find it really entertaining and awesome like that they’re learning these skills so young. So much fun” - “His parents had mentioned that he had come a long way as well with all of those things and he was enjoying the experience of having all those different things to do out there [playground]” - “They love them [loose parts]” |
| OLPP increased knowledge/learning about physical activity | - “Well, the other kids were playing…so the other kids were like watching them, like what are they doing and then some of them came over to try it….” - “He was kind of manager of the project. And they were all helping, and it was a very collaborative effort…” - “In fact, some of the other children would come over and take their hand and show them around and then you could see the confidence building each time they went until eventually they could do it themselves. So, it was really nice to see how they worked together and were mentoring each other and cooperating and helping the younger ones, so that they could grow their gross motor skills too, so I think that was one big thing, advantage of the whole program right because they, the younger ones learned from the older ones” - “They really used a lot of cognitive skills – you could really see the wheels turning, and their problem solving through the whole thing, really thinking outside the box as to how am I going to make this work?” - “They know what they want when they go outside. They know what they’re going for and they run for it to make sure they get it” |
| OLPP contributed to increased cognitive and social development | - “Teamwork, and turn taking, and encouraging each other, sharing ideas, um, building confidence through the whole thing. You know, really learning, um, independence –play and, um, learning to, uh – or coach each other for what to do next and then feed from each other” - “Oh, just when it was higher like I think the crates falling over. They’re pretty lightweight, they’re fairly easy to carry so and they’re pretty good with telling their friends to watch it, we need more space” - “Like my group of four-year-olds collectively decided where’s the path, where is it too high, like my, like the class independently decided that’s too high, we should not jump from here, and they did that independently cause they jumped off there like that hurts my feet when I land, that was their risk assessment” - “…cause they would give directions, so they really understood the ways they had to balance you know heavy and light and what would make one go up and the other go down, like they understood that process, they didn’t use the words exactly but you can tell that they knew what they had to do” - “Well, that’s just, like they don’t look for us nearly as much outside as they do inside” |
